# Genome-wide analysis of the VQ motif-containing gene family and expression profiles during phytohormones and abiotic stresses in wheat (*Triticum aestivum* L.)

**DOI:** 10.1186/s12864-022-08519-3

**Published:** 2022-04-11

**Authors:** Lili Zhang, Keke Wang, Yuxuan Han, Luyu Yan, Yan Zheng, Zhenzhen Bi, Xin Zhang, Xiaohong Zhang, Donghong Min

**Affiliations:** 1 College of Agronomy, State Key Laboratory of Crop Stress Biology for Arid Areas, Northwest A&F University, Shaanxi Yangling, China; 2 College of Life Sciences, Northwest A&F University, Yangling, Shaanxi China

**Keywords:** VQ gene family, Wheat, Expression pattern, Plant hormones, Abiotic stress

## Abstract

**Background:**

VQ motif-containing (VQ) proteins are cofactors of transcriptional regulation that are widely involved in plant growth and development and respond to various stresses. The VQ gene family has been identified and characterized for many plants, but there is little research on VQ gene family proteins in wheat (*Triticum aestivum* L.).

**Results:**

In this study, 113 *TaVQ* genes (40 homoeologous groups) were identified in the wheat genome. TaVQ proteins all contain the conserved motif FxxhVQxhTG, and most of the *TaVQ* genes do not contain introns. Phylogenetic analysis demonstrated that TaVQ proteins can be divided into 8 subgroups (I-VIII). The chromosomal location mapping analysis indicated that *TaVQ* genes are disproportionally distributed on 21 wheat chromosomes. Gene duplication analysis revealed that segmental duplication significantly contributes to the expansion of the TaVQ gene family. Gene expression analysis demonstrated that the expression pattern of *TaVQ* genes varies in different tissues. The results of quantitative real-time PCR (qRT-PCR) found that *TaVQ* genes displayed different expression levels under different phytohormones and abiotic stresses. The *cis*-elements analysis of the promoter region demonstrated that stress responses, hormone responses, growth and development, and WRKY binding elements are all widely distributed. Additionally, a potential regulatory network between TaVQ proteins and WRKY transcription factors was visualized.

**Conclusion:**

This study systematically analyzed the wheat TaVQ gene family, providing a reference for further functional characterization of *TaVQ* genes in wheat.

**Supplementary Information:**

The online version contains supplementary material available at 10.1186/s12864-022-08519-3.

## Background

Common wheat (*Triticum aestivum* L.) is one of the most widely planted and important food crops in the world. It is also the primary food crop in China. However, various biotic and abiotic stresses severely restrict the quality and production of wheat [1–3]. Over their evolutionary history, plants have formed a signal transmission network in response to external stresses. In response to abiotic stress, plant transcription factors such as WRKY, MYB, NAC, AP2/ERF, bHLH, and bZIP interact with the *cis*-acting elements in the promoters of stress-responsive genes to regulate the activation or inhibition of target genes, which improves plant resistance to stress [4–6]. The WRKY family is one of the largest transcription families and plays a vital role in plant response to various abiotic stresses, growth, and development [7]. The WRKY domain binding specifically to the *cis*-acting element W-box (T)(T)TGAC(C/T) of the target gene, which are participated in stress response and signaling [6, 8]. Studies have shown that the promoter regions of many VQ genes contain W-Box *cis*-acting elements, which can form complexes with WRKY transcription factors to play a vital regulatory role in plant vegetative growth, differentiation, seed development, and stress response [9, 10].

VQ proteins constitute an ancient family of transcriptional regulators, and their members are widely distributed among various species [11, 12]. VQ proteins constitute highly conserved proteins with a short FxxhVQxhTG (x: any amino acid h: hydrophobic amino acid) amino acid sequence motif. They interact with WRKY transcription factors via conserved V and Q residues [13]. The proteins were further categorized based on the terminal three amino acids in the conserved motif. For example, six types of motifs were identified in *Arabidopsis* (VTG, LTS, FTG, YTG, LTG, LTD) [13]. VQ motif-containing proteins were first identified in *Arabidopsis* by Morikawa et al. using a yeast two hybrid-assay [14]. The VQ protein family has since been identified in many plants: 34, 40, 74, 61, and 18 members of the VQ protein gene family have been found in *Arabidopsis*, rice, soybean, maize, and grapevine, respectively [13, 15–17]. While VQ proteins were thought to be transcriptional regulators only found in plants, Jiang et al. found VQ proteins in some fungi, lower animals, and bacteria, indicating that the *VQ* gene family is widely distributed [12].

Typically, VQ proteins interact with the family of WRKY transcription factors to mediate stress response and growth and development in plants. For example, *Arabidopsis* IKU1/AtVQ14 regulates endosperm development and seed size by interacting with MINI3/ WRKY10 [18]. VQ20 interacts with WRKY2 and WKRY34 and regulates pollen development in *Arabidopsis* [19]. VQ29 physically interacts with PIF1 and plays an important role in early seedling development. [20]. Many studies have demonstrated that VQ proteins play a vital role in plant response to abiotic stresses. The AtVQ9 protein acts as a repressor of the WRKY8 transcription factor and interacts with WRKY8 to regulate the Na ^+^ /K ^+^ ion balance of plants under high salt stress [21]. *AtCaMBP25* (*VQ15*) expression is induced in *Arabidopsis* seedlings by osmotic stress. *Arabidopsis* overexpressing *AtCaMBP25* is more sensitive to osmotic stresses during seed germination and seedling growth. This demonstrates that AtCaMBP25 is a negative regulator of salt stress in *Arabidopsis* [22]. Studies have also demonstrated that VQ proteins are involved in plant response to pathogen infections. VQ12 and VQ29 interact with themselves and each other and participate in the jasmonic acid (JA)-mediated signaling pathway to negatively regulate the resistance of plants to *Botrytis cinerea* [23]; *JASMONATE-ASSOCIATED VQ-MOTIF GENE1* (*JAV1/VQ22*) is a key gene in the JA signaling pathway and serves as a repressor protein during JA-mediated defense against herbivorous insects and necrotic pathogens [24, 25]. The overexpression line of *BnMKS1* (*BnVQ7*) in *Brassica napus* was more resistant to *Leptosphaeria maculans* infections at the adult stage [26].

To date, research on plant VQ proteins has mostly been performed on model plants. There is little information about the VQ protein gene family in wheat and how it controls plant growth and development and responds to various biotic and abiotic stresses. As such, we performed a comprehensive analysis of the genome sequence data of common wheat and identified 113 members of the VQ protein gene family. We analyzed their phylogeny, conserved motifs, gene structure, and promoters. To better understand the potential functions of the VQ protein gene family in wheat, we performed gene expression analysis on various plant hormones, biotic and abiotic stresses. Our results provide a basis for identifying and classifying *TaVQ* genes. Additional studies will contribute to a better understanding of how TaVQs functions are involved in plant stress response and growth.

## Results

### Identification and characterization of *TaVQ* genes in wheat

**Fig. 1:**
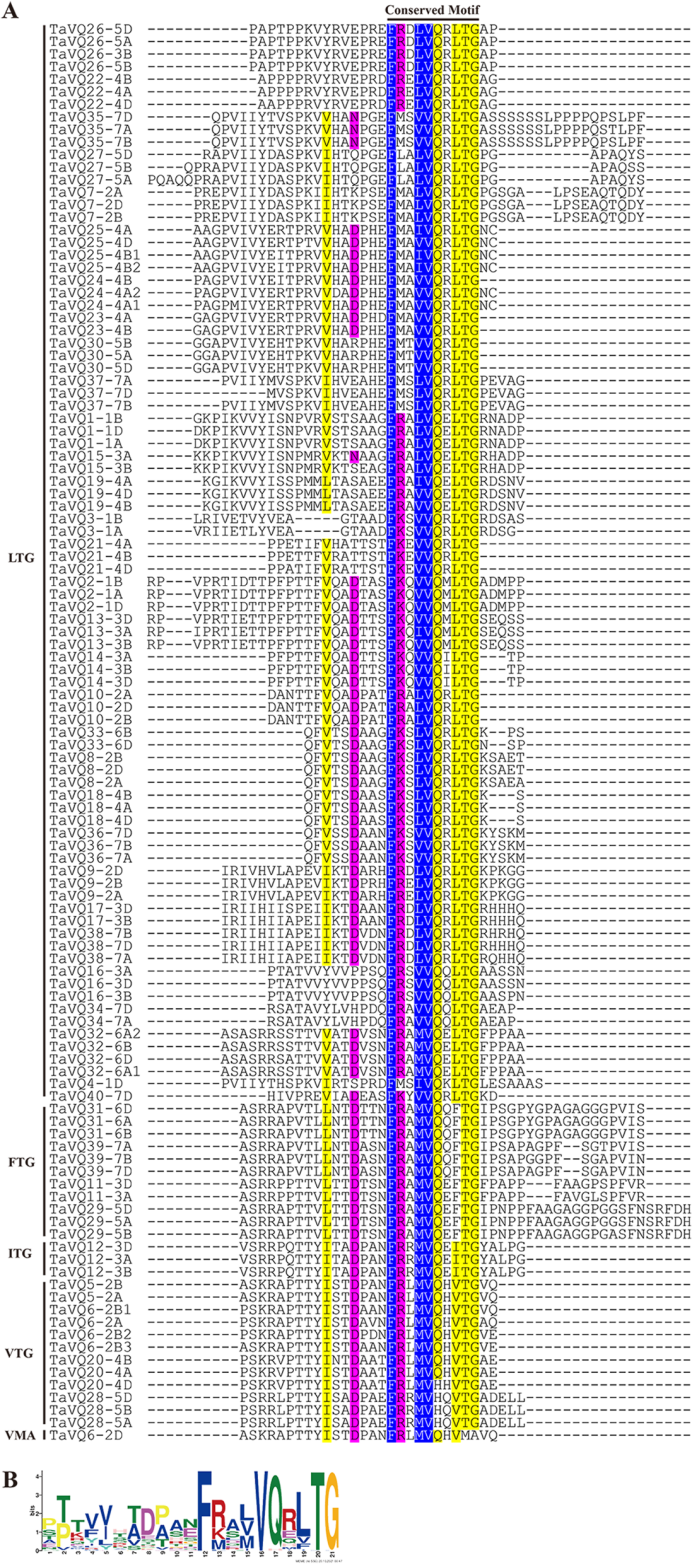
Multiple sequence alignment of VQ proteins in wheat. **A** Multiple sequence alignment of VQ domain of 113 VQ proteins in wheat. Amino acids that are conserved throughout are shaded in different colors. **B** Conserved motif of TaVQ proteins.

The Hidden Markov Model (HMM) of the VQ motif (PF05678) was used to search the wheat protein database for putative VQs. We identified a total of 113 candidate genes encoding VQ proteins in the wheat genome, which were named *TaVQ1*-*TaVQ40* based on the location of their encoding gene on the chromosomes. The subgenome location and Chromosome numbers were accounted for by ensuring that the gene names, for example, *TaVQ1-1A* (Fig. 1 and Additional file 2: Table S1). Two or three inparalogs belonging to the same genome were distinguished by consecutively numbered (e.g. *TaVQ6-2B1*, *TaVQ6-2B2*, *TaVQ6-2B3*). Results of the protein sequence alignment demonstrated that of the 113 proteins, 86 contained the conserved motif FxxxVQxLTG and 26 contained FxxxVQxxTG. The conservative motif of TaVQ6-2D is FxxxVQxVMA, which was not reported in previous studies. We also found that the core amino acids of the four proteins (TaVQ20-4D, TaVQ28-5A, TaVQ28-5B, and TaVQ28-5D) were VH instead of VQ, which is similar to OsVQ37 and OsVQ39 in rice (Fig. 1) [27]. These 113 VQ proteins have different physiological and biochemical properties; their amino acid length ranges from 80 to 573 amino acids, with an average of 190 amino acids (Additional file 2: Table S1). The molecular weight of these VQ proteins varied from 8.26 to 59.36 kDa and have a theoretical isoelectric point (PI) ranging from 5.08 to 11.33, 69.0% (78/113) of TaVQ protein members were basic nature. The instability index of 113 TaVQ proteins ranged from 22.53 (TaVQ18-4A) to 86.84 (TaVQ10-2D), 85.8% (97/113) were found unstable while only the remaining 14.2% (16/113) TaVQ proteins had stable nature at the level of biological sequences. The aliphatic index is a feature of thermostability that ranged from 44.3 (TaVQ29-5A) to 78.66 (TaVQ21-4A), of which 59.3% (67/113) less than 65 indicates that most TaVQ protein members are not thermostability. The predicted Grand Average of Hydropathy (GRAVY) of TaVQ proteins ranged from 0.116 (TaVQ20-4A) to -0.821(TaVQ15-3A) suggesting the hydrophilic nature of 97.35% TaVQ proteins. The subcellular localization of TaVQ proteins was predicted: most of them (108/113) is located in the nucleus, two are located in the cell membrane/nucleus (TaVQ40-7D, TaVQ31-6D), one is located on the cell wall/chloroplast/nucleus (TaVQ16-3B), one is located in the chloroplast/nucleus (TaVQ16-3D), and one is located in the cell membrane (TaVQ33-6B).

### Phylogenetic analysis in wheat VQ proteins

**Fig. 2:**
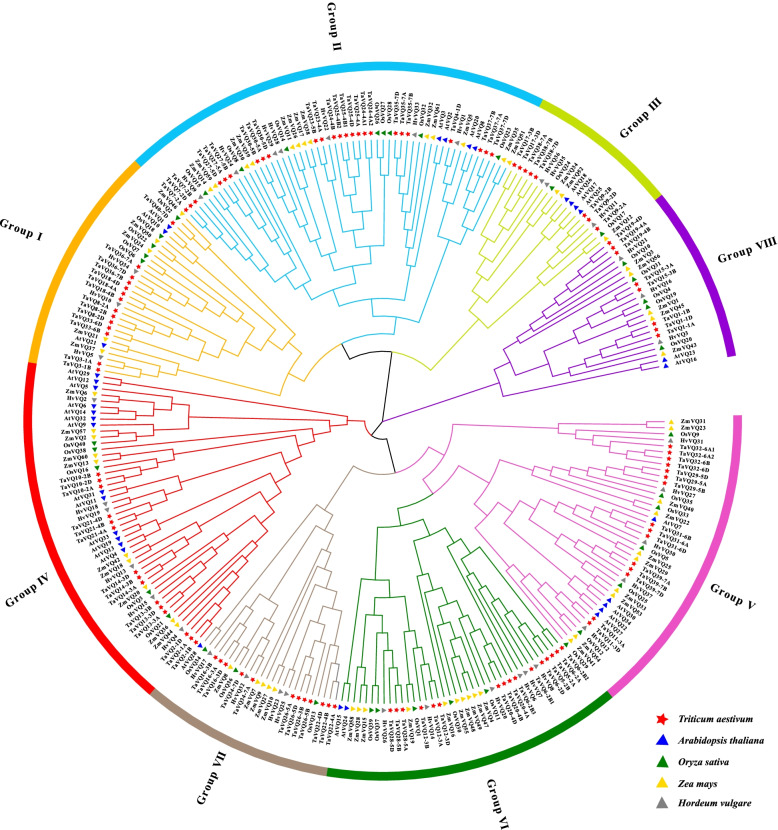
Phylogenetic tree of VQ proteins from wheat, *Arabidopsis thaliana, Oryza sativa*, *Zea mays*, and *Hordeum vulgare* constructed using the neighbor-joining method in MEGA 7. 113 TaVQ proteins, 34 AtVQ proteins, 40 OsVQ proteins, 61 ZmVQ proteins and 37 HvVQ proteins are clustered into eight subgroups (I-VIII). Proteins from wheat, *Arabidopsis*, rice, maize, and barley are indicated by red stars, blue triangles, green triangles, yellow triangles, and grey triangles, respectively. Details of the VQ proteins from *Arabidopsis*, rice, maize, and barley are listed in Additional file 3: Table S2

**Fig. 3:**
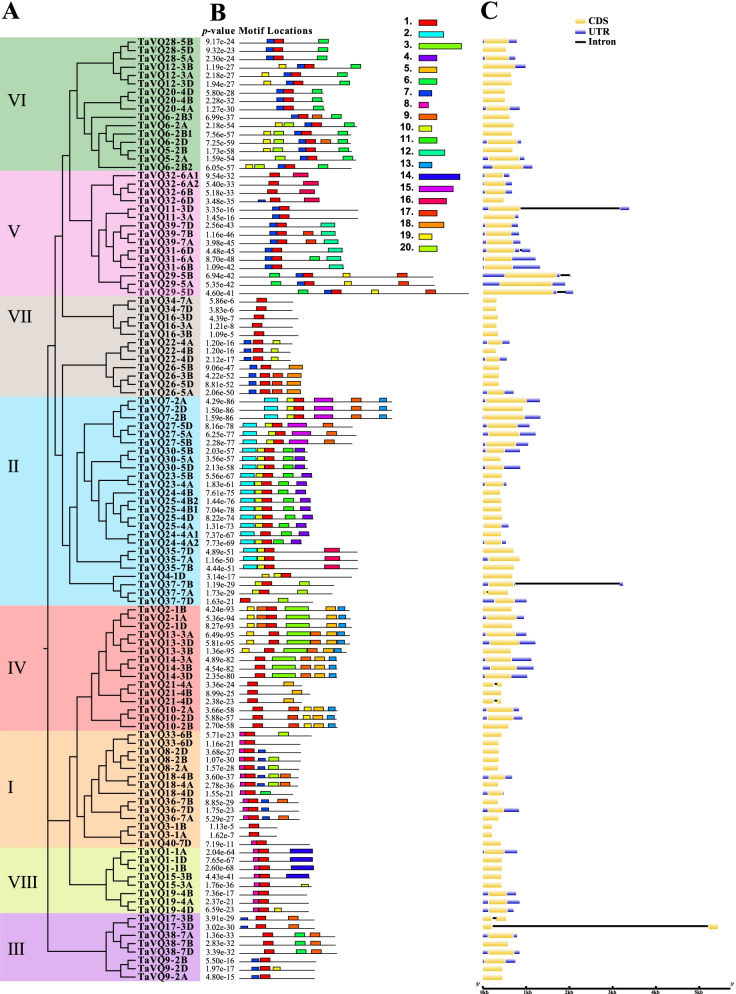
Phylogenetic analysis, conserved motifs, and gene structure of VQ in wheat. **A** Phylogenetic tree of 113 VQ proteins in wheat constructed based on the results of sequence alignment. The tree was created with 1000 bootstraps using the neighbor-joining (NJ) method in MEGA7. **B** Motif compositions of 20 conserved motifs of the VQ protein in wheat were identified by the MEME tool. Each specific motif is indicated by a different colored box; detailed motif information is shown in Additional file 4: Table S3. **C** Gene structure of the *VQ* genes in wheat from the GSDS database. Exons are indicated by yellow rectangles, UTR is indicated by blue rectangles, and lines connecting two exons represent introns. Box and line length are displayed proportionally to gene length

To explore the phylogenetic relationship of the VQ proteins in wheat (113), rice (40), *Arabidopsis* (34), barley (37), and maize (61), a phylogenetic tree was constructed using the Mega 7.0 (Fig. 2). Detailed information about rice, *Arabidopsis*, barley, and maize *VQ* genes are shown in Additional file 3: Table S2. We also constructed a second phylogenetic tree with only the 113 wheat VQ proteins (Fig. 3A). The VQ proteins are clustered into eight distinct groups (group I-VIII) according to the structural characteristics of the VQ protein sequences and previous classification of VQ proteins from *Arabidopsis* and rice [27]. Of these eight groups, group II member sizes were significantly larger (25 TaVQ proteins); group VIII and III only contains eight TaVQ proteins respectively, and there are 14, 15, 15, 16, 12 TaVQ proteins in groups I, IV, V, VI, VII respectively. Compared to the VQ proteins of *Arabidopsis* and rice, the clade clustering pattern of TaVQ (Fig. 2) only slightly differs from previous studies in *Arabidopsis* and rice [27]. The evolutionary relationships demonstrate that TaVQ proteins have a closer relationship with gramineous barley, rice, and maize, while TaVQ proteins and a distant relationship with dicotyledon *Arabidopsis* VQ proteins in the same group.

### Conserved motifs and gene structural analysis of TaVQ

The sequences of the TaVQ proteins were aligned, and a phylogenetic tree was built (Fig. 3A). The conserved motifs were predicted, while 20 motifs were determined using the MEME tool. Detailed motif information is displayed in Additional file 4: Table S3. Results demonstrated that each TaVQ protein has between one and six conserved motifs (Fig. 3B). All proteins have motif 1, which has a specialty VQ domain. Members from the same group share similar motifs, while some motifs are found only in one group: motif 12 is found only in group V, motif 14 is found only in group VIII, motif 3 and motif 5 are found only in group IV, and motifs 2, 4, 10, and 15 are only found in group II (Fig. 3B). TaVQ proteins in the same group have similar motifs, which aligns with the results of the phylogenetic analysis. This indicates that proteins in the same group perform similar functions.

Exon–intron distribution was analyzed using the GSDS online tool (http://gsds.cbi.pku.edu.ch) (Fig. 3C) to better outline the structural features of *VQ* genes in wheat. This analysis determined that 91.15% (103/113) of *TaVQ* genes do not contain introns, while the other 10 genes have only one intron. This could be due to the loss of introns in *VQ* genes during its evolutionary history.

### Genome distribution and gene duplication of *TaVQ* genes

**Fig. 4:**
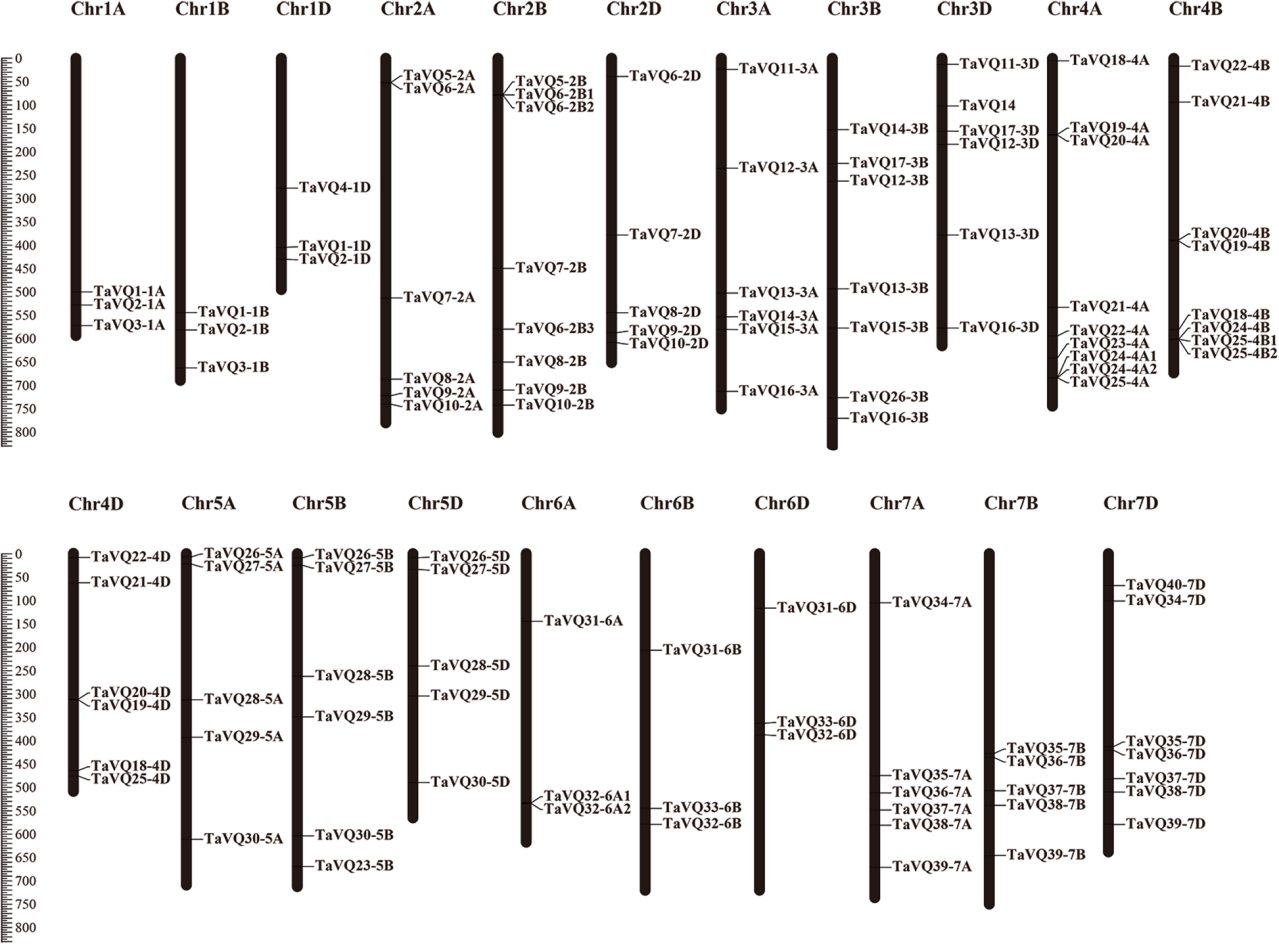
The distribution of 113 *VQ* genes across chromosomes of wheat. Chromosome numbers are listed above chromosomes, while chromosome size is listed, in megabases (Mb), on the left side of the figure

The chromosomal locations were mapped to further investigate genetic differences in the TaVQ gene family. MapChart software was used to produce the chromosome map of *TaVQ* genes on 21 chromosomes according to chromosome location information of *TaVQ* genes found in the GFF3 file. Members of the same subgenome are typically distributed at similar locations in homoeologous chromosomes. *TaVQ* genes were distributed among 21 chromosomes, but are not evenly distributed on different chromosomes. Of them, nine *TaVQ* genes are located on chromosome 4A, eight *TaVQ* genes are located on chromosomes 2B and 4B, and three *TaVQ* genes are located on homologous group 2 and group 6 (Fig. 4). This uneven distribution indicates that *TaVQ* gene duplication events could have occurred in 2B, 4A, and 4B chromosomes during wheat evolution. We also analyzed the homologous group of *TaVQ* genes (Table 1). Almost two-thirds of all wheat *VQ* genes were found in triplets (66.4%): three *TaVQs* localized on the three homologous groups (A:B:D) shared high homology (1:1:1). The proportion of triplets in the TaVQ gene family exceeded the proportion of homologous triplets in the entire wheat genome. This high proportion of homologous triplets could be the primary cause of the expansion of the TaVQ family. The percentage of *VQ* genes with homeolog-specific duplications (n:1:1/1:n:1/1:1:n) was higher compared to all wheat genes (15.0% vs 5.7%), and the ratio of orphans/singletons was lower than the whole wheat genome (1.8% vs 37.1). However, the loss ratio of a single homeolog in a *TaVQ* gene (1:1:0/1:0:1/0:1:1) was similar to that of the whole wheat genome (14.2% vs 13.2%; Table 1).

**Table 1:** Homologous group in the wheat TaVQ gene family

Homoeologous group (A:B:D)	All wheat genes (%)^a^	Number of TaVQs groups	Number of TaVQs	% of total TaVQs
1:1:1	35.8	25	75	66.4
n:1:1/1:n:1/1:1^b^	5.7	4	17	15.0
1:1:0/1:0:1/0:1:1	13.2	8	16	14.2
Other ratios	8.0	1	3	2.7
Orphans/singlets	37.1	2	2	1.8
Total	99.8	40	113	100.0

^a^According to IWGSC(2018)

^b^n > 1

**Fig. 5:**
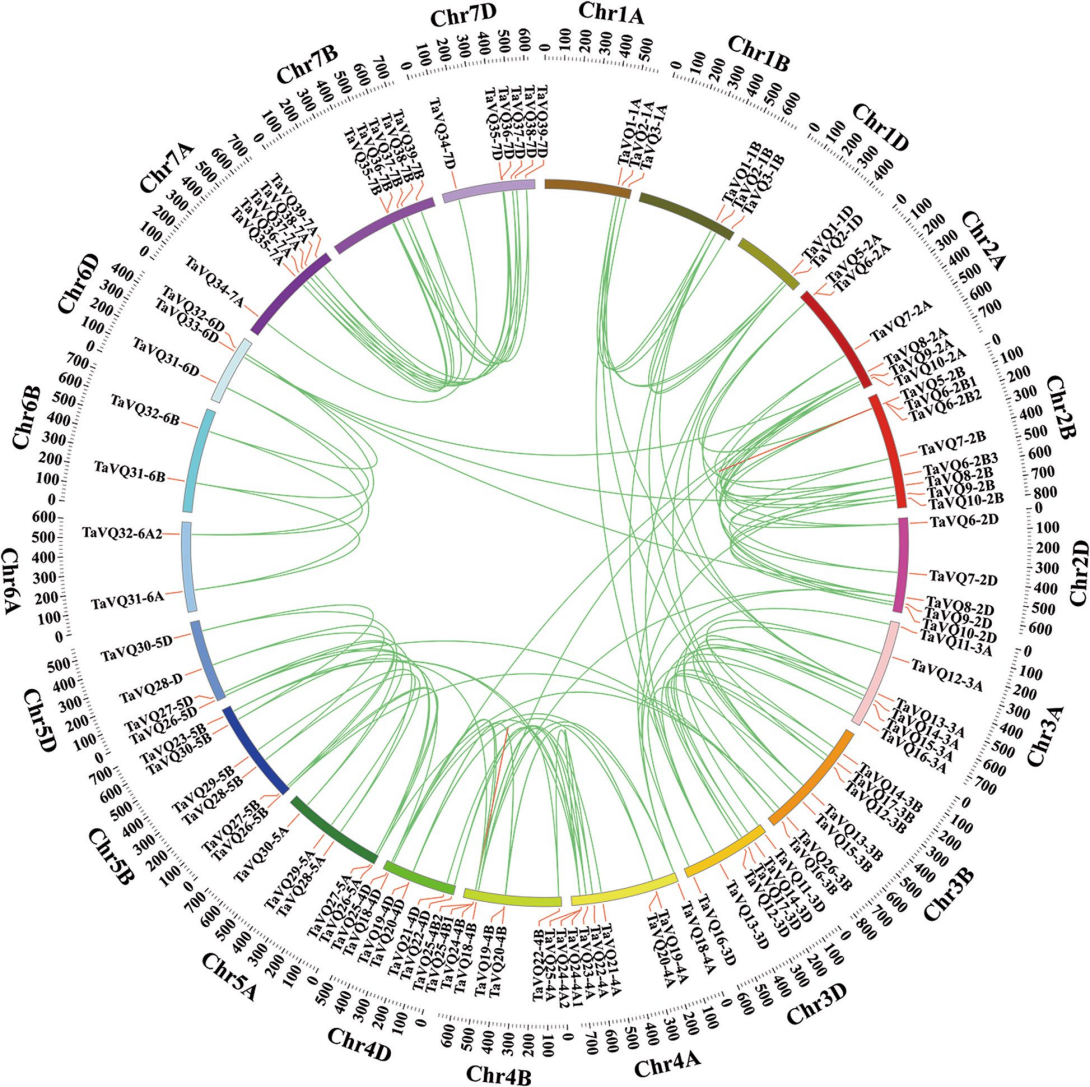
Analysis of duplication events of *VQ* genes in wheat. Chromosome numbers are indicated at the outer edge of the circle, while the scale represents megabases (Mb). Segmental duplication gene pairs are linked by green lines, and tandem repeated genes are linked by red lines

Gene duplication is an important process contributing to genomic evolution and is an important factor in gene family expansion. It is primarily divided into tandem duplication and segmental duplication. We found that 94 pairs (104 genes) are homologous genes and 43 pairs (31 genes) are segmental duplication genes distributed on different chromosomes (Fig. 5). We next analyzed tandem duplication events occurring within the wheat VQ gene family and found two triple-duplication *TaVQ* genes located on chromosome 2B (*TaVQ5-2B*, *TaVQ6-2B1*, *TaVQ6-2B2*) and chromosome 4B (*TaVQ24-4B*, *TaVQ25-4B1*, *TaVQ25-4B2*) (Fig. 5). These results suggest that tandem and segmental duplication events are necessary to expand the TaVQ gene family. Segmental duplication could have played a significant role in this process.

The Ka/Ks ratio indicates whether there is selective pressure on duplication events. The ratio of Ka/Ks > 1 typically represents positive selection and a ratio equal to 1 shows neutral selection. However, a ratio < 1, indicates the presence of a purifying selection effect [28]. We calculated the Ka/Ks values of duplication *VQ* genes pairs in wheat to understand the extent and nature of this selection pressure. Our results demonstrated that the Ka/Ks ratio of most duplicated *TaVQ* gene pairs is less than 1, ranging from 0.005 to 1.608 with an average of 0.330 (Additional file 5: Table S4). Of them, 138 duplicated pairs had a Ka/Ks ratio < 1, which suggests that *TaVQ* genes have undergone strong purifying selection. Moreover, five pairs (*TaVQ5-2A*/*TaVQ6-2B3*, *TaVQ6-2B3*/*TaVQ6-2B2*, *TaVQ6-2A*/*TaVQ6-2D*, *TaVQ6-2A/TaVQ6-2B1*, and *TaVQ18-4A*/*TaVQ18-4B*) were higher than 1.00, which shows the presence of positive selection pressure (Additional file 5: Table S4).

### Prediction of SSR and miRNAs targeting *TaVQ* genes

We detected 28 simple sequence repeats (SSRs) in 25 of 113 *TaVQ* genes. The detailed information of the SSRs was shown in the Additional file 6: Table S5. Discovered gene-specific SSRs were divided into 3 types: mononucleotides, dinucleotides, and trinucleotides. Among all the identified SSRs, a single SSR was detected in most *TaVQ* genes except for *TaVQ29-5A*, *TaVQ29-5D*, and *TaVQ4-1D* each carrying two SSRs. In addition, *TaVQ29-5A*, *TaVQ31-6B*, *TaVQ31-6D*, and *TaVQ39-7A* carried each a composite SSR. Trinucleotide repeats (75.0%) outnumbered the other repeats followed by dinucleotide repeats (7.1%). In the future, the SSRs in *TaVQ* genes after due validation may be utilized for developing markers to be used for wheat breeding.

To understand the function of *TaVQ* genes, we predicted putative miRNA target sites in 113 *TaVQ* genes using the psRNATarget server. The results showed that 38 *TaVQ* genes are targeted by 15 putative miRNAs. The regulatory network showed that one miRNA can target more than one *TaVQ* gene (Additional file 1: Figure S1). For instance, tae-miR9780 target 9 *TaVQ* genes, tae-miR5384-3p target 7 *TaVQ* genes, and tae-miR9676-5p and tae-miR9677b target 6 *TaVQ* genes. These miRNAs belong to different miRNA families such as miR160, miR395, miR1130, and miR9657. Studies have shown that these miRNA families play a key role response to various biotic and abiotic stresses [29–31].

### Expression patterns of *TaVQ* genes

**Fig. 6:**
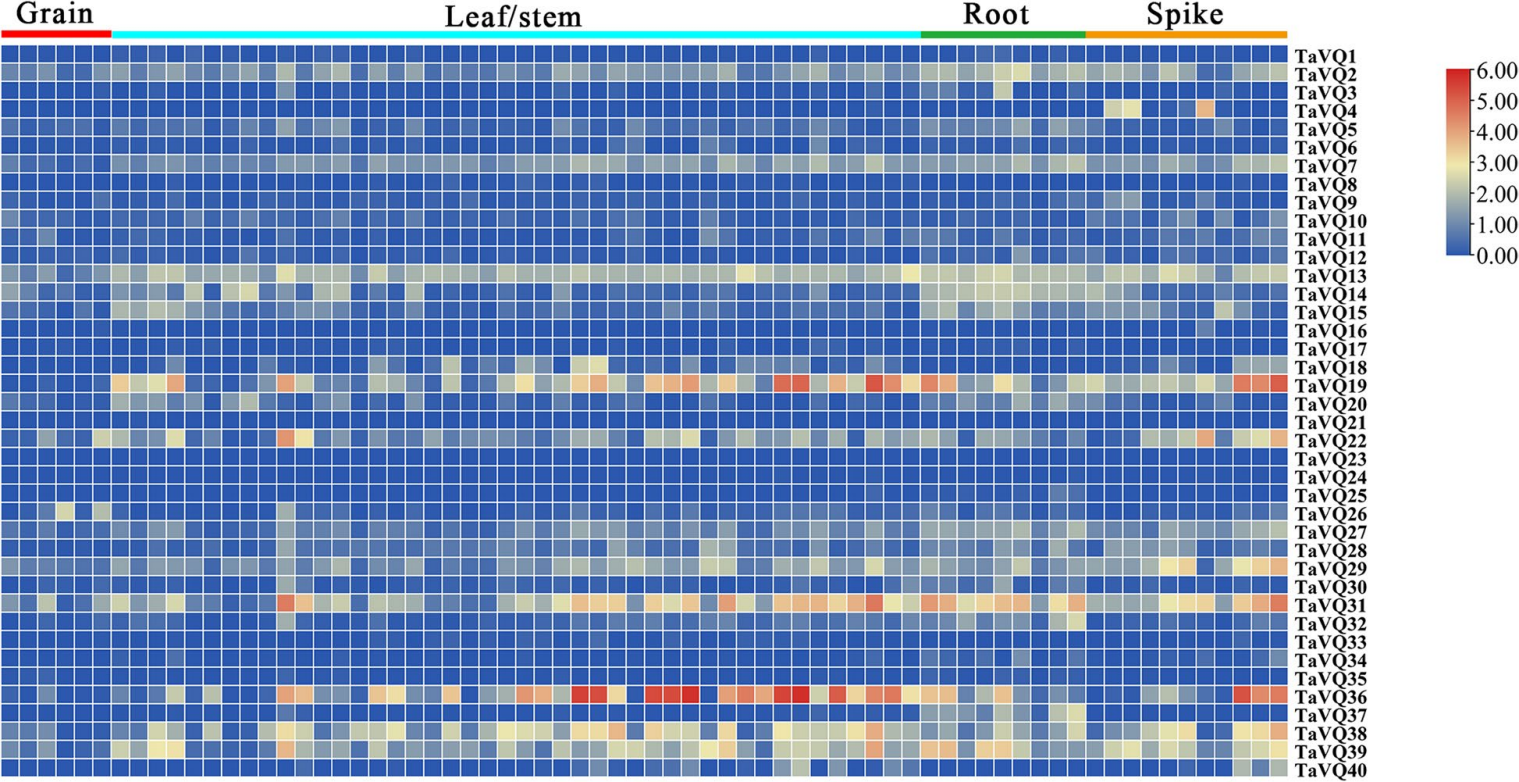
*TaVQ* gene expression during wheat development. Expression values of wheat *VQ* genes were obtained using RNA-seq data [29]. Heatmap displays *VQ* gene expression levels across developmental stages and tissues. Heatmap was generated using log2 (TPM + 1) values. Tissues and TPM values are respectively displayed in Additional file 7: Table S6 and Additional file 8: Table S7

We analyzed the expression levels of 113 *TaVQ* genes using available RNA-seq data from previous research to identify the patterns of the spatial and temporal expression of *VQ* genes in wheat [32]. Based on the TPM values, we found that some members of the TaVQ gene family are expressed in only one organ, while some were expressed more broadly. Detailed TPM values are shown in Additional file 7: Table S6 and Additional file 8: Table S7. Of the 40 *VQ* homologous genes, 75% (30/40) were detected in at least one developmental stage, with a wide range of expression levels and a tpm value ranging from 1.0 to 55.0. The remaining 25% (10/40) of *TaVQ* genes showed very low expression levels, while tpm < 1 was considered unexpressed. Some *TaVQ* genes were differentially expressed in the various stages of development in four organs. For 45 leaf/stem tissues, 24 *TaVQ* genes were expressed in at least one leaf/stem tissue, while 18 genes were not expressed in roots and spikes (Fig. 6). Only 25% of genes were expressed in the six stages of grain development with relatively low transcriptional abundances and expression levels ranging from 1.0 to 4.6 (Fig. 6 and Additional file 8: Table S7). Some genes are specifically expressed in only one organ. For example, *TaVQ6* and *TaVQ11* were only expressed in the leaf/stems, *TaVQ12* and *TaVQ37* were only expressed in the roots, and *TaVQ4*, *TaVQ9*, and *TaVQ10* were only expressed in the spikes. In general, the overwhelming majority of *TaVQ* genes were not expressed or only a few genes were expressed with low expression levels in grains, while more than half of *TaVQ* genes were expressed in leaf/stems, roots, and spikes. This suggests that these *VQ* genes play different roles when regulating wheat growth and development.

### Expression of *TaVQ* genes under abiotic stress

**Fig. 7:**
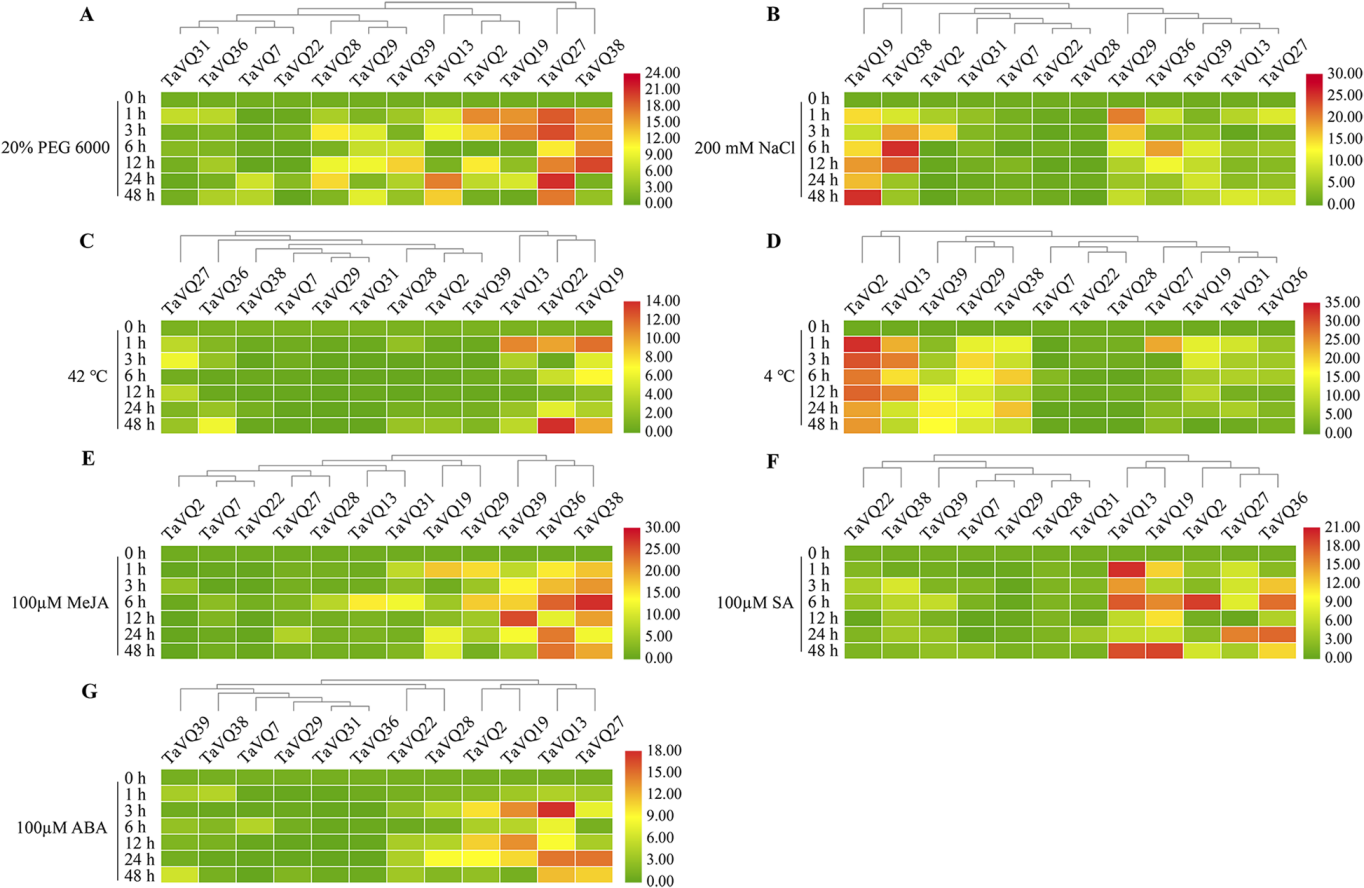
Heatmap of 12 wheat *VQ* genes expression profiles in seedlings under **A** PEG 6000, **B** NaCl, **C** 42℃, **D** 4℃, **E** methyl jasmonate (MeJA), **F** salicylic acid (SA), and **G** abscisic acid (ABA) based on qRT-PCR. The 2^−∆∆Ct^ method was used to calculate relative expression levels, while TBtools (v1.068) was used to generate the heatmap

This study seeks to better understand *VQ* gene expression levels in wheat under different abiotic stresses, including drought (PEG 6000), salt (NaCl), low temperature (LT, 4℃), high temperature (HT, 42℃), and phytohormones such as methyl jasmonate (MeJA), salicylic acid (SA) and abscisic acid (ABA). Therefore, we selected 12 genes from the TaVQ gene family that were expressed at different developmental stages in four tissues and subjected them to qRT-PCR analysis. Our results demonstrated that the 12 *TaVQ* genes displayed distinctly different transcriptional responses and presented a complex regulatory mechanism under multiple abiotic stresses or phytohormone treatments (Fig. 7). Transcript levels of these 12 *TaVQ* genes were different from transcriptional levels under osmotic stress induced by 20% PEG 6000. The four *TaVQ* genes (*TaVQ2*, *TaVQ19*, *TaVQ27*, and *TaVQ38*) were rapidly and significantly up-regulated in the early stage (1 h) of 20% PEG 6000 treatment, while their expression levels were up-regulated more than 15-fold compared to the controls (Fig. 7A). Of them, the expression levels of *TaVQ27* and *TaVQ38* were continuously up-regulated until they peaked at 24 h, and 12 h, respectively. Conversely, the expression of *TaVQ2* and *TaVQ19* increased after 1 h of drought stress, then gradually decreased and finally dropped to the lowest point after 48 h of drought stress treatment. In the salt stress treatment, the 12 *TaVQ* genes clustered into three groups based on the various expression patterns (Fig. 7B). The first group contained 2 genes (*TaVQ19* and *TaVQ38*) that were significantly up-regulated (> 15-fold), while expression levels peaked at 48 h and 6 h, respectively. The second group contained five genes (*TaVQ2*, *TaVQ31*, *TaVQ7*, *TaVQ22*, and *TaVQ28*) with down-regulated or up-regulated transcriptional expression. The third group consisted of five genes (*TaVQ29*, *TaVQ36*, *TaVQ39*, *TaVQ13*, and *TaVQ27*) that were up-regulated in response to salt stress (Fig. 7B). Following HT stress treatment, the transcript levels of the 12 *TaVQ* genes were mostly down-regulated or slightly up-regulated, except for *TaVQ13*, *TaVQ22*, and *TaVQ19*, which were significantly up-regulated (> 10-fold) (Fig. 7C). When the seedlings were treated with LT, the expression patterns of these 12 genes were classified into four groups (Fig. 7D). The first group consists of two genes (*TaVQ2* and *TaVQ13*) with up-regulated expression levels of 36.884 and 26.511 that peaked at 1 h and 3 h, respectively. The second group contained three genes (*TaVQ39*, *TaVQ29*, and *TaVQ38*) in which the transcription levels gradually increased and peaked under extended LT stress. The third group consisted of three genes (*TaVQ7*, *TaVQ22*, and *TaVQ28*) in which the expression was down-regulated or slightly up-regulated. The fourth group contained found genes (*TaVQ27*, *TaVQ19*, *TaVQ31*, and *TaVQ36*) that showed early responses to LT stress, though their expression gradually decreased as the stress time increased (Fig. 7D).

The expression patterns of 12 *TaVQ* genes following exogenous phytohormone (MeJA, SA, and ABA) treatments were further analyzed. *TaVQ* gene expression after MeJA treatments was grouped into three categories: slightly up-regulated or down-regulated transcripts (*TaVQ2*, *TaVQ7*, *TaVQ22*, *TaVQ27*, *TaVQ28*, *TaVQ13*, and *TaVQ31*), increased expression at 1 h followed by a gradual decrease (*TaVQ19* and *TaVQ29*), or continuously increased transcript levels (*TaVQ39*, *TaVQ36*, and *TaVQ38*) (Fig. 7E). Expression levels of the 12 *TaVQ* genes were analyzed in seedlings treated with SA. As shown in Fig. 7F, five *TaVQ* genes (*TaVQ13*, *TaVQ19*, *TaVQ2*, *TaVQ27*, and *TaVQ36*) significantly responded to SA treatments. Gene expression analysis to ABA for 12 *TaVQ* genes showed greater differences in expression (Fig. 7G). Compared to the control, the transcript abundances of four *TaVQ* genes (*TaVQ2*, *TaVQ19*, *TaVQ13*, and *TaVQ27*) were up-regulated at all ABA treatment time points. These results demonstrate that *TaVQ* genes are involved in wheat response to multiple abiotic stresses and phytohormones, and have complex response mechanisms due to their functional differentiation.

### The modulatory network between TaVQ proteins with TaWRKY transcription factors

**Fig. 8:**
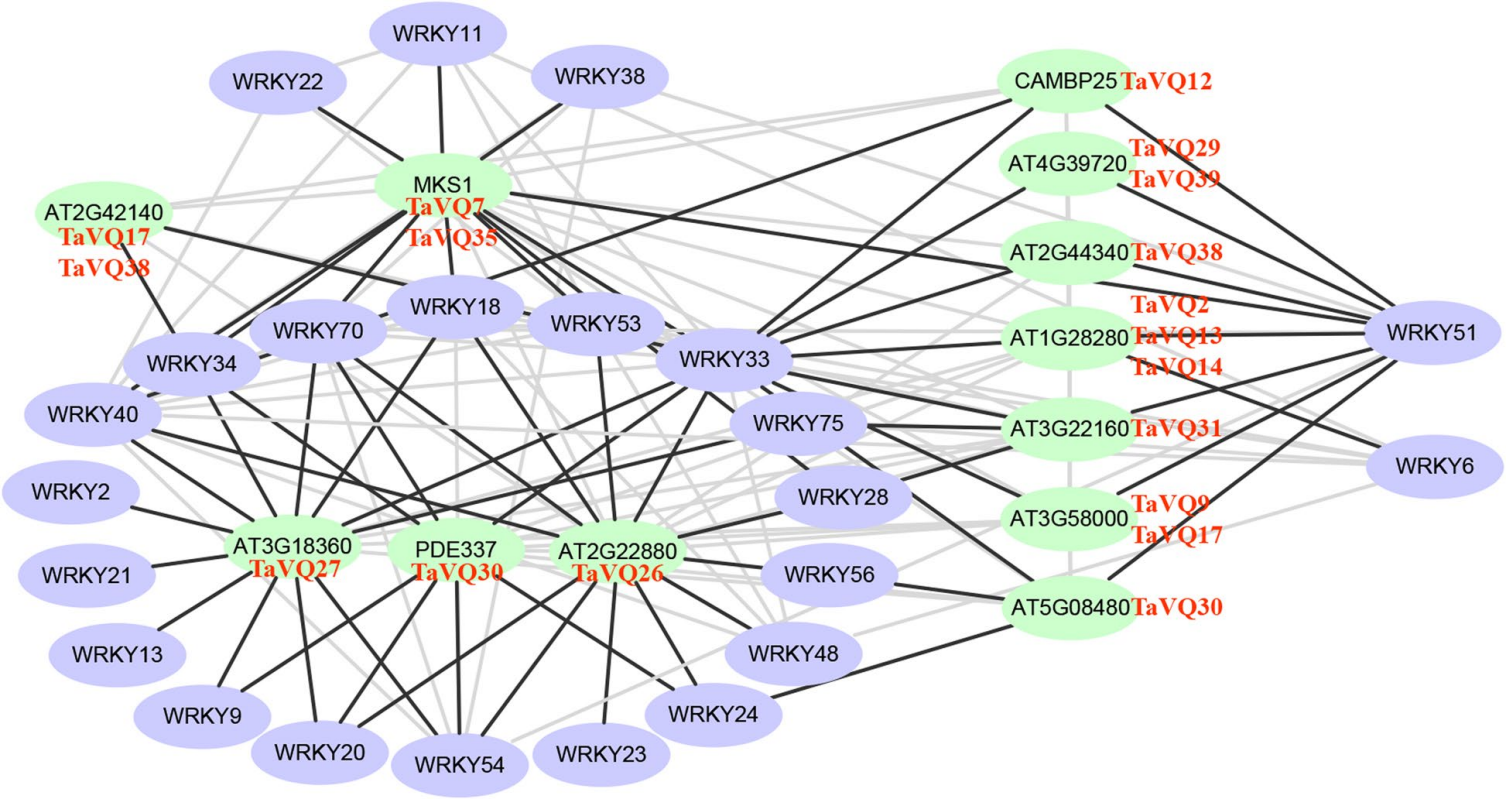
Interaction network of TaVQ proteins with WRKY transcription factors. The prediction of interacting network models between TaVQ proteins and WRKY proteins was performed using the STRING online database and the interaction network was drawn in Cytoscape 3.7.1. Black and gray lines indicate the interaction of a VQ protein and a WRKY protein, and two VQ proteins or two WRKY proteins, respectively. Homologous genes in wheat and *Arabidopsis* are displayed in red and black, respectively.

To better understand the functional and potential interactions of VQ proteins in wheat, a modulatory network was generated by constructing an *Arabidopsis* association model using STRING software. Studies have demonstrated that VQ proteins can form complexes with WRKY transcription factors and play an important role in plant growth, differentiation, seed development, and stress [9]. As shown in Fig. 8, 15 TaVQ proteins were homologous with *Arabidopsis* proteins and interacted with 23 WRKY transcription factors. These 15 TaVQ proteins all interact with at least two WRKY transcription factors (Fig. 8). MKS1 (TaVQ7, TaVQ35), AT3G18360 (TaVQ27), PDE337 (TaVQ30), and AT2G22880 (TaVQ26) interacted with at least seven WRKY transcription factors to form a key node. Additionally, WRKY 51 and WRKY33 closely interact with multiple VQ proteins, forming a complex regulatory network. Similar to some WRKY, most TaVQ proteins interact with other members of TaVQ proteins. Therefore, TaVQ proteins likely require interaction with VQ proteins to function, however, the specific purpose of the interaction between VQ proteins and WRKY transcription factors in wheat requires further study.

### Analysis of *cis*-elements in the promoters of the *TaVQ* genes

**Fig. 9:**
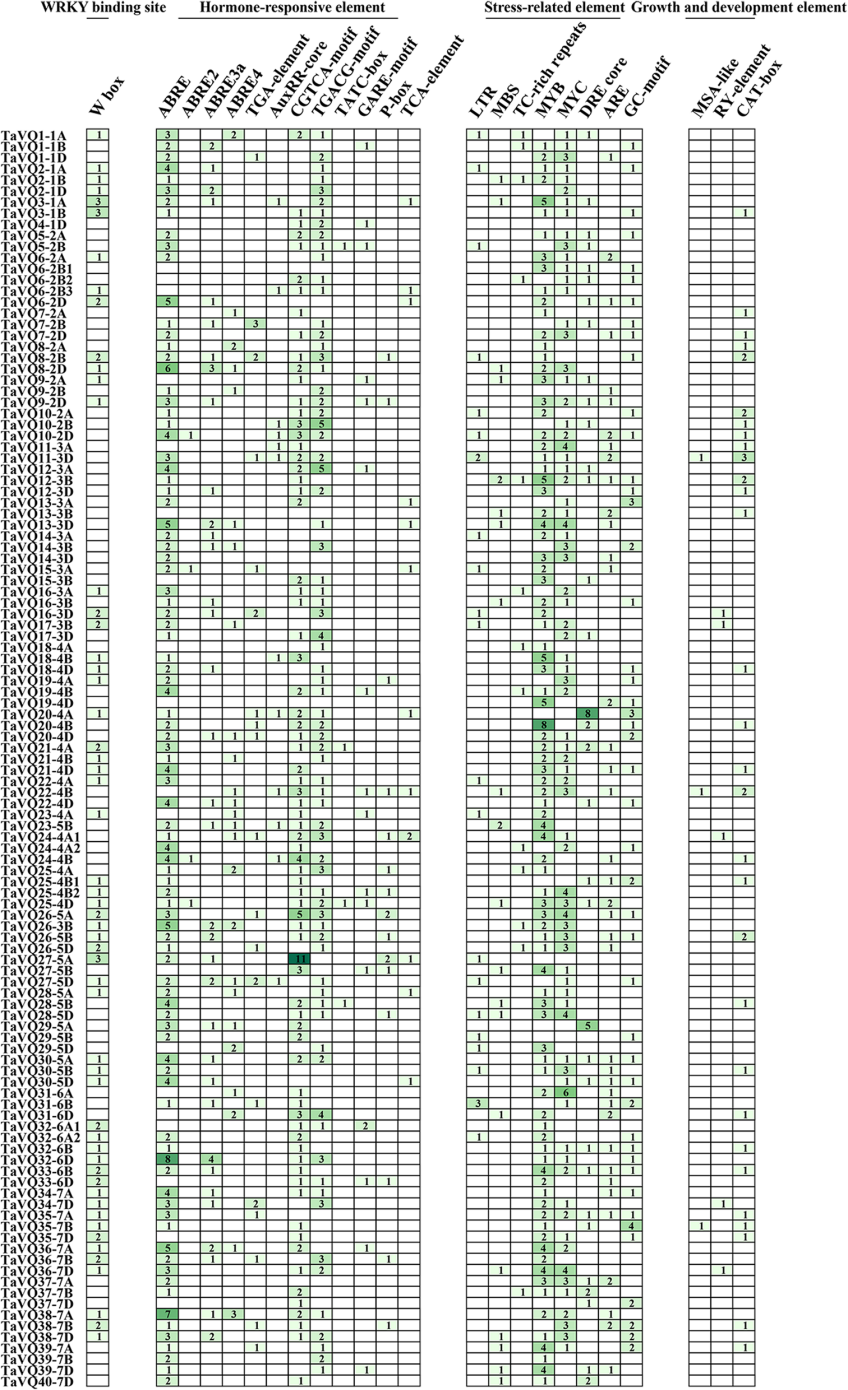
Number of each *cis*-acting element of the *TaVQ* gene promoter region (1.5 kb upstream of the translation start site)

To explore the function and regulatory mechanism of these *VQ* genes in wheat, the WRKY binding site (W-box), hormone-responsive elements (ABRE, TGA-element, AuxRR-core, CGTCA-motif, TGACG-motif, TATC-box, GARE-motif, P-box, and TCA-element), stress-related regulatory elements (LTR, MBS, TC-rich repeats, MYB, MYC, DRE core, ARE, and GC-motif), and growth and development elements (MSA-like, RY-element, and CAT-box) in the promoter region were searched by the PlantCARE database. The WRKY transcription factor can specifically bind to the (T)(T)TGAC(C/T) sequence (W-box) to regulate the target gene expression that contains the W-box elements in the promoter, which then participates in various physiological and biochemical responses in plants [33]. We found that W-box *cis*-elements were distributed in the promoter region of nearly half (55/113) of *TaVQ* genes, indicating that WRKY proteins could bind to *VQ* genes and respond to environmental stimuli (Fig. 9 and Additional file 9: Table S8).

Hormone-responsive *cis*-elements include *cis*-acting elements involved in ABA response elements (ABRE), auxin-responsive elements (TGA-element, AuxRR-core), *cis*-acting regulatory elements involved in MeJA response elements (CGTCA-motif, TGACG-motif), *cis*-acting element involved in SA response elements(TCA-element), and gibberellin-responsive elements (TATC-box, GARE-motif, P-box). Of these, *cis*-acting elements involved in MeJA response were abundant in the *TaVQ* gene promoter region, while 88.5% (100/113) of *TaVQ* genes contained at least one MeJA response element in their promoter regions (Fig. 9 and Additional file 9: Table S8). *Cis*-acting regulatory elements involved in ABA response were also present in the *TaVQ* gene promoter region, while 86.7% (98/113) promotors of the family members at least contained one ABA-responsive element.

Stress-related regulatory elements were widely distributed in the promoter region of *TaVQ* genes. Most *TaVQ* members contained one or more MYB and MYC elements in their promoters, which are *cis*-acting elements involved in stress-induced drought, salt, and ABA responses. Additionally, other response elements related to stress were also detected, including low-temperature (LTR), defense and stress (TC-rich repeats), anaerobic (ARE), anoxic (GC-motif), MYB binding sites involved in drought-inducibility (MBS), and DREB binding sites involved in drought, salt, low temperature, and ABA responses (DRE core) (Fig. 9 and Additional file 9: Table S8).

Elements related to plant growth and development were detected, including MSA-like (cell cycle regulation element), RY-element (seed-specific regulation element), and CAT-box (related to meristem expression). Of all *TaVQ* genes, 31% (35/113) contained at least one element related to plant growth and development in their promoter region, indicating that these *TaVQ* genes could participate in wheat growth and development (Fig. 9 and Additional file 9: Table S8). These results indicate that *TaVQ* genes could interact with WRKY transcription factors during plant stress tolerance and growth and development.

## Discussion

VQ proteins are an ancient family that is primarily involved in signal pathways to regulate plant growth, development, and response to various biotic and abiotic stresses by interacting with partners such as WRKYs and MAPKs [11, 27, 34]. VQ gene families have been identified in various plant species, including *Arabidopsis* [13], rice [27], maize [17], soybean [35], tobacco [36], and tea plant [37]. However, *VQ* genes remain largely uncharacterized in wheat, though the whole genome of hexaploid wheat has been sequenced. Therefore, genome-wide analyses of wheat *VQ* genes can be performed by analyzing their bioinformatics and expression patterns to further understand their regulation under various abiotic and phytohormonal treatments. This could establish a foundation for further functional characterization of VQ proteins.

The VQ protein has a highly conserved VQ motif FxxhVQxhTG (x: any amino acid h: hydrophobic amino acid), while the amino acid sequences of other regions are diverse. Based on residue differences, this study found six types of the VQ conserved motif of the 113 TaVQ proteins: FxxxVQxLTG (86/113), FxxxVQxFTG (11/113), FxxxVQxITG (3/113), FxxxVQxVTG (8/113), FxxxVHxVTG (4/113), and FxxxVQxVMA (1/113). Previous studies have found four kinds of VQ motifs in rice (ITG, LTG, VTG, and FTG) [27], six kinds of VQ motifs in Chinese cabbage (LTG, YTG, VTG, FTG, LTV, and LTS) [38], five kinds of VQ motifs in soybean (LTG, FTG, VTG, LTR, and LTS) [35], five kinds of VQ motifs in maize (LTG, VTG, ATG, ITG, and FTG) [17], and six kinds of VQ motifs in *Arabidopsis* (LTG, LTS, LTD FTG, VTG, and YTG) [13]. Additionally, there is a unique VQ conserved domain type (VMA) in wheat that is not present in other plant species (Fig. 1). Therefore, the different types and numbers of VQ motif variations in different species could be related to functional differences in VQ gene family members. The core amino acids of TaVQ20-4D, TaVQ28-5A, TaVQ28-5B, and TaVQ28-5D were VH instead of VQ, which is similar to OsVQ37 and OsVQ39 in rice and ZmVQ15, ZmVQ28, and ZmVQ58 in maize [17, 27]. The VQ core domain changed to VH only in monocotyledonous plants, which could be due to differences in monocotyledons and dicotyledons during their evolutionary history. Additionally, the minimum VQ protein in wheat is 80 amino acids (TaVQ3-1A), with an average length of 190 amino acids. This is similar to most reported VQ proteins in plants, which are less than 300 amino acids. In previous studies, the localization prediction analysis of VQ proteins demonstrated that most VQ proteins are located in the nucleus, while some are found in the cytoplasm, mitochondria, and chloroplasts [13, 27, 37]. Our study found that 95.6% of TaVQ proteins were located in the nucleus, while two genes are located in the cell membrane/nucleus, one in the cell wall/chloroplast/nucleus, one in the chloroplast/nucleus, and one on the cell membrane (Additional file 2: Table S1). These results are consistent with observations of the VQ protein in many known plants, including *Arabidopsis* *thaliana* [13], poplar [39], and tea trees [37].

Previous studies have found that VQ proteins of different species have different evolutionary histories and are phylogenetically clustered into different groups, though there are no reports on this classification [12]. Our results demonstrate that TaVQ proteins were clustered into eight groups according to the phylogenetic tree. While they share a close evolutionary relationship with VQ proteins in monocot rice, they are more distantly related to the dicot *Arabidopsis*. This indicates that TaVQs are highly conserved during the evolutionary history of plants. We also found that the TaVQ proteins among members of the same group have similar conserved motifs, indicating that TaVQ proteins within the same group have similar functions. Previous studies have confirmed that most VQ proteins in plants have no introns, for example, 88.2%, 92.5%, 88.5%, 92.3%, 79.6%, and 86.2% of the *VQ* genes in *Arabidopsis*, rice, maize, tomato, apple, and *Moso bamboo* did not contain introns, respectively [13, 17, 27, 40–42]. This could be because plant *VQ* genes have lost a large number of introns during their evolutionary history.

Gene duplication events are an important part of genomic evolution and are the primary driver of species evolution [43, 44]. Gene duplication has two modes: segmental and tandem gene duplication, both of which play important roles in expanding certain gene families in the plant genome [45, 46]. In this study, we analyzed the contribution of segmental and tandem gene duplication to the expansion of the VQ gene family in wheat. A total of 31 segmental gene replication events and two cases of three gene tandem arrangements were identified, indicating that segmental duplication events play a major role in expanding the wheat VQ gene family. Jiang et al. conducted gene duplication analysis of *VQ* genes in 12 species (*Brachypodium distachyon*, *Sorghum bicolor*, *Brassicarapa bicolor*, *Solanum lycopersicum*, *Oryza sativa*, *Zea mays*, *Glycine max*, *Selaginella moellendorffii*, *Setaria italica*, *Arabidopsis thaliana*, *Populus trichocarpa*, and *Physcomitrella patens*) and demonstrated that segment duplication is the primary mechanism of *VQ* gene family amplification [12]. During these duplication events, the original gene performs the ancestral function while the new gene takes on a new function [47, 48]. We also calculated the Ka/Ks ratio, which can determine whether the protein-coding gene has selection pressure. Our results demonstrate that the Ka/Ks ratio of most *TaVQ* duplication gene pairs was less than 1, indicating that most *TaVQ* genes experienced effective purifying selection. The calculated Ka/Ks ratio of five *TaVQ* duplication gene pairs exceeded 1.00, indicating positive selection pressures. This analysis indicates that purification selection plays an important role in the evolution of the TaVQ gene family.

Many studies have demonstrated that VQ proteins are involved in the regulation of plant growth and development. The *vq8* dysfunction mutant in *Arabidopsis* showed a light green and slow growth phenotype, indicating that VQ8 plays a key role in the growth and development of *Arabidopsis* [13]. Various studies have found that the AtVQ14 (HAIKU1; IKU1) protein is expressed in early endosperm and can promote endosperm development. The *atvq14* mutant has reduced endosperm growth and smaller seeds, indicating that AtVQ14 plays an important role in the regulation of early endosperm development and *Arabidopsis* seed size [18, 49]. Additionally, the overexpression of *AtVQ17*, *AtVQ18*, and *AtVQ22* severely impaired plant growth and development, indicating that these three VQ proteins could inhibit plant growth [13]. However, the flowering time of *AtVQ29* overexpression plants was significantly delayed, and the hypocotyl growth of *AtVQ29* transgenic *Arabidopsis* was less sensitive to far-red and low-intensity white light [13, 20]. VQ20 is specifically expressed in pollen and can interact with WRKY2 and WKRY34 to affect pollen development and function [19]. The heterologous expression of *MKS1* (*ATVQ21*) causes dwarfing and delayed flowering in *Kalanchoë blossfeldiana* and *Petunia hybrids* [50]. Additionally, the transcription level of *VQ* genes in other plants has demonstrated significant histological specificity. For example, 24 soybean *VQ* genes were relatively highly expressed in nine tissues [35], while 14 *Moso bamboo VQ* genes were highly expressed in leaf, panicle1, panicle2, root, and rhizome tissues [42]. Most *VQ* genes in the tea plant were differentially expressed in the roots, stems, leaves, and flowers [37]. We also used publicly available RNA-seq data to analyze the tissue expression of 40 *VQ* homologous genes in wheat and found that most of the *TaVQ* genes were relatively highly expressed in leaf/stem, roots, and spikes, but less so in grains. These results demonstrate that members of the VQ gene family are widely involved in plant growth and development.

Many VQ motif-containing genes are involved in abiotic stress responses, such as drought, salt, high temperature, low temperature, and ABA, all of which regulate plant growth [16, 17, 27, 38, 40–42, 51]. *AtVQ9* was strongly expressed under NaCl treatment, and the *atvq9* mutant showed a high germination rate and low electrical conductivity under salt stress. Therefore, VQ9 negatively regulates *Arabidopsis* tolerance to salt stress [21]. *AtCaMBP25* (*AtVQ15*) gene expression was induced in *Arabidopsis* seedlings under dehydration, low temperature, or high salt stress. *Arabidopsis* with overexpression of *AtCaMBP25* showed higher sensitivity to osmotic stress of NaCl and mannitol during seed germination and seedling growth. Therefore, AtCaMBP25 is a negative regulator of salt stress in *Arabidopsis* and participates in the stress signal transduction pathway [22]. *Arabidopsis* VQ18 and VQ26 interact with ABI5 transcription factors to form protein complexes, which negatively regulate ABA signal transduction and promote seed germination [52]. *IbVQ4* expression was induced by drought and salt treatment, while IbWRKY2 can interact with IbVQ4 to regulate abiotic stress tolerance in plants [53]. PeVQ28 positively regulates the salt tolerance of transgenic *Arabidopsis* through an ABA-dependent signaling pathway. Compared with the wild type, *Arabidopsis* overexpressing *PeVQ28* showed increased resistance to salt stress, with lower malondialdehyde and higher proline contents. The expression of salt and ABA response genes in *PeVQ28* overexpressed *Arabidopsis* also increased under salt stress [54]. Overexpression of the *MdVQ37* gene in apple plants significantly reduced tolerance, while the enzyme activity and photosynthetic capacity of the transgenic lines decreased under drought stress compared with the wild type [55]. Ding et al. identified 26 VQ family genes in tomato, of which *SlVQ6* has the highest expression in tissues and organs. Additional studies found that *SlVQ6* overexpression decreased the heat tolerance of *Arabidopsis* and down-regulated the expression of stress response-related genes [40]. In addition, the VQ gene family also responds to different abiotic stresses in maize, tobacco, tea plant, and *Eucalyptus grandis* [17, 36, 37, 56]. In this study, we detected the expression levels of 12 *TaVQ* genes under drought, salt, high-temperature, low-temperature, and ABA stress. We found that *TaVQ* genes showed different expression trends under different abiotic stresses. This indicates that the response mechanism of *TaVQ* genes to abiotic stresses is complex and diverse.

Previous evidence has demonstrated that the expression of VQ family genes is regulated by biotic stress and by the MeJA and SA hormones. SA and JA are important defense signaling molecules that play an important role in the regulation of different pathogens [57]. For example, MaVQ5 physically interacts with MaWRKY26 and represses MaWRKY26 in activating jasmonic acid (JA) biosynthesis, and attenuates the transactivation of the MAWRKY26-induced JA biosynthesis genes *MaLOX2*, *MaAOS3*, and *MaOPR3* [58]. *Arabidopsis VQ12* and *VQ29* genes were strongly induced by JA and *Botrytis cinerea*. Pathogen resistance of the *vq29* mutant and *amiR-vq12 vq29* double mutant was significantly enhanced, while the transgenic plants of *VQ12* and *VQ29* were sensitive to the pathogen. This indicates that VQ12 and VQ29 negatively regulate plant resistance to the pathogen [23]. *Arabidopsis JAV1/VQ22* is a key gene in the JA signaling pathway and acts as a negative regulator of JA-mediated plant defense [21, 24]. Overexpression of Apple *MdVQ37* reduces basal thermotolerance by regulating multiple transcription factors and SA homeostasis [59]. MKS1 (AtVQ21) interacts with WRKY25 and WRKY33 to regulate the expression of the SA-related defense gene *PR1* [60]. The expression levels of VQ family genes in tobacco, soybean, and *Eucalyptus grandis* under different hormone treatments also indicated that *VQ* genes were involved in the regulation of various plant hormones [35, 36, 56]. We detected the expression patterns of 12 *TaVQ* genes under MeJA and SA treatments and found that certain *TaVQ* genes are highly expressed after MeJA and SA treatments. Our research demonstrates that *TaVQ* genes could be involved in the regulation of JA and SA signaling pathways.

As a transcriptional regulatory cofactor, VQ proteins can interact with several proteins to participate in the regulation of various physiological and biochemical processes in plants. Of them, interactions with the WRKY transcription factor are the most important function of VQ proteins. For example, WRKY57 and WRKY33 interact with SIB1 and SIB2 and regulate the expression of JAZ1 and JAZ5, which are key inhibitors of the JA signal pathway. This blocks the JA signal and weakens the resistance of WRKY33 to *B.cinerea* [61, 62]. Recent studies have demonstrated that SIB1 and SIB2 can interact with WRKY75 to form a complex and negatively regulate ABA-mediated leaf senescence and seed germination [63]. *Arabidopsis* AtVQ15 can interact with AtWRKY25 and AtWRKY51 to negatively regulate osmotic stress [13, 22]. VQ20 interacts with WRKY34 and WKRY2 to regulate the expression of several pollen development-related genes in plant male gametogenesis to participate in the regulation of *Arabidopsis* male gametes [19]. In this study, we found that multiple TaVQ proteins can interact with different WRKY transcription factors. Analysis of the *cis*-elements demonstrated that nearly half of TaVQ genes contain one or more W-box *cis*-elements (WRKY transcription factors specific binding elements) in their promoter regions. These results indicate that WRKY transcription factors could play an important role in the function of VQ proteins.

## Conclusions

This study identified a total of 113 wheat VQ motif-containing genes and performed a comprehensive genome-wide study on *TaVQ* genes. This included genome-wide identification, phylogenetic analysis, chromosome distribution, *cis*-acting elements, and expression pattern analysis. *TaVQ* gene expression patterns in different tissues indicate that they play an important role in wheat growth and development. Expression analysis of *TaVQ* genes under biotic and abiotic stress indicates that *TaVQ* genes are involved in the regulation of wheat biotic and abiotic stress. The interaction network between TaVQ proteins and TaWRKY transcription factors demonstrated that certain TaVQ proteins must interact with TaWRKY transcription factors to function. Our results provide a foundation for future study of the function of TaVQ proteins in wheat.

## Methods

### Identification of the *TaVQ* gene family in wheat

Wheat protein sequences were downloaded from the EnsemblePlants [64], while Hidden Markov Model (HMM) profiles of the VQ domains (PF05678) were downloaded from the Pfam database [65]. The wheat proteins database was then searched with the HMM profiles using the hmmsearch tool of HMMER software (v3.3.1) with cut-off values (e-value) of 1e-5. All candidate VQ motif-containing sequences were filtered to keep only VQ domains, which were then confirmed by the National Center for Biotechnology Information (NCBI) Conserved Domain Database [66], the Pfam database, and the SMART database [67]. Bioinformatics analysis of each *VQ* gene was performed, and the length (amino acids), the pI (isoelectric point), and MW (molecular weight) were obtained from the online ExPasy website [68].

### Phylogenetic analysis

Thirty-four *Arabidopsis* VQ protein sequences were downloaded from the TAIR database [69], 40 rice and 61 maize VQ protein sequences were downloaded from the Phytozome database [70], 37 barley VQ protein sequences were downloaded from the EnsemblePlants. Multiple sequence alignments of the amino acid sequences were performed using Clustalw2 with default parameters. Based on the alignment files, the MEGA7.0 software was used to construct a phylogenetic tree using the neighbor-joining method [71]. The bootstrap test method of the phylogenetic tree was used with 1000 replicates for each node; other parameters were default. The phylogenetic tree was then drawn using EVOLVIEW [72].

### Gene structure construction, protein domain, and motif analysis

We investigated the exon–intron structures of wheat *VQ* genes based on the information obtained from the GFF files using the online Gene Structure Display Server [73]. To evaluate the structural divergence of wheat VQ proteins, all 113 VQ full-length amino acid sequences were identified using the Multiple EM for Motif Elicitation (MEME) online tool [74]. The parameters were as follows: the number of motifs was 20 with zero or one occurrence per sequence and a motif width between 6 and 50 residues.

### Chromosome locations and gene duplication

The start and end location information of 113 wheat *VQ* genes were obtained from URGI [75], while the chromosomal location of the *TaVQ* gene distribution was drawn with MapChart 2.3 software[76]. All *TaVQ* nucleotide sequences were aligned using BLASTN software with an E-value below 1e-20 [77]. We used the following criteria to analyze *VQ* gene duplication events in wheat: (1) length of the alignable sequence covered > 75% of the longer gene; (2) similarity of the aligned region > 75% at the nucleotide level [78]. Duplicated *VQ* gene pairs in wheat were obtained and visualized using Circos-0.69 software [79]. The nonsynonymous substitution rate (Ka), the synonymous substitution rate (Ks), and the Ka/Ks ratio of orthologous *VQ* gene pairs were obtained using the KaKs_calculator2.0 [80].

### Prediction of SSR and miRNAs targeting *TaVQ* genes

Simple sequence repeats (SSRs) were developed within the genomic sequences of *TaVQ* genes using MISA [81]. The SSR searching parameters were: mononucleotides ≥ 10, dinucleotides ≥ 6, trinucleotides ≥ 5, tetranucleotides ≥ 5, pentanucleotides ≥ 5, and hexanucleotides ≥ 5. The psRNATarget server was used to predict the potential miRNAs and their targets in *TaVQ* genes using default parameters [82]. Interaction networks between targeting miRNAs and *TaVQ* genes were drawn using the Cytoscape software (version 3.7.1) [83].

### Expression profiling of *TaVQ* genes

To study *TaVQ* gene expression in different organs, RNA-seq data of wheat *VQ* genes based on developmental time-course was downloaded from the Wheat Expression Browser [32]. Seventy tissues/time points from wheat cv *Azhurnaya* were considered as developmental stages [32]. Transcripts per million (tpm) were extracted as *VQ* gene expression values and an average of the homologs. Heatmaps were constructed using TBtools (v1.068) [84] on log_2_tpm + 1 for 40 *VQ* homologous genes from 113 *VQ* genes.

### *Cis*-elements in the promoter regions of *TaVQ* genes

To better understand the family of *TaVQ* genes, we investigated the *cis*-elements of the *TaVQ* gene promoters. We analyzed upstream sequences within 1,500 base pairs (bp) of the coding sequences (CDS) for promoter analysis. The sequences were submitted to the PlantCARE database [85] to identify their *cis*-elements. The number of *cis*-acting elements of the *TaVQ* genes was displayed using TBtools (v1.068).

### Plant materials and stress treatments

The wheat used in this study was Chinese spring, which was planted in an artificial chamber with stable temperatures of 23–25℃ at a 16 h light/8 h dark cycle. Abiotic stress treatments were as follows: treatment of one-week-old seedlings with 4 °C (low-temperature stress), 42 °C (high-temperature stress), 200 mM NaCl, and 20% (w/v) polyethylene glycol 6000 (PEG 6000), respectively, as described previously [86, 87]. For phytohormone treatments, wheat was treated with 100 µM abscisic acid (ABA), 100 µM methyl jasmonate (MeJA), and 100 µM salicylic acid (SA) [88]. Both the control and treated seedlings were harvested at 0 h, 1 h, 3 h, 6 h, 12 h, 24 h, and 48 h after treatment. Samples from all three biological replicates were immediately frozen in liquid nitrogen at -80 °C for subsequent RNA extraction.

### RNA extraction and qRT-PCR analysis of *TaVQ* genes

RNAiso Plus (TaKaRa, Japan) was used to extract total RNA from Chinese spring leaves during the three-leaf period, according to the manufacturer’s instructions. The first-strand cDNAs were synthesized using EasyScript® One-Step gDNA Removal and cDNA Synthesis SuperMix (Transgen Biotech, China) according to the manufacturer’s instructions, and methods in the previous study [89, 90]. Subsequently, qRT-PCR was performed with a QuantStudio 7 Flex real-time PCR system (Life Technologies, USA) using PerfectStart™ Green qPCR SuperMix (Transgen Biotech, China) according to the manufacturer’s instructions as mentioned before[89]. Primers used in the qRT-PCR analysis were designed by the Primer 5.0 software, which is listed in Additional file 10: Table S9. The β-actin was used as a housekeeping gene for the normalization of gene expression in at least three biological replicates. The 2^−△△Ct^ method was used to calculate relative expression levels, while TBtools (v1.068) was used to visualize the heat maps of gene expression.

### Interaction network analysis of TaVQ proteins

Protein sequences of the wheat WRKY family were obtained from the wheat genome database. Protein sequences of wheat TaVQ and TaWRKY transcription factors were mapped into the *Arabidopsis* database by constructing an *Arabidopsis* association model. The STRING online database (version 11.5) [91] was used to predict the TaVQ protein and TaWRKY interaction network model with a combined score of > 0.4. Interaction networks between TaVQs and TaWRKYs were then drawn using the Cytoscape software.

## Supplementary Information

**Additional file1:** **Fig S1.** Interaction network of targetd miRNAs and theirTaVQ partners using Cytoscape 

**Additional file2:** **Table S1.** The detailed information of 113 TaVQ genes identified in wheat genome.

**Additional file3: Table S2. **The VQ genes identified from Arabidopsis, rice, barley , and maize in this study. 

**Additional file4: Table S3.**The amino acid sequences of 20 putative motifs.

**Additional file5:** **Table S4.** Ka/Ks values of duplicated TaVQ gene pairs

**Additional file6:** **Table S5.** Predicted gene specific SSR markers in genomic sequences of wheat TaVQ genes.

**Additional file7: ** **Table S6.** Tissues and stages for expression analysis for Fig. 6 and Table S7.

**Additional file8:** **Table S7.** RNA-seq data analysis of TaVQ genes expression profiling in different tissues.

**Additional file9:** **Table S8.** All the cis-regulatory elements of 113 TaVQ gene promoters predicted by PlantCARE online tool.

**Additional file10:** **Table S9.** The primers used for qRT-PCR in this study.

## Data Availability

All data generated or analyzed during this study are included within the article and its additional files. The RNA-Seq data involved in this study were downloaded from the Wheat Expression Browser (http://www.wheat-expression.com/).

## Abbreviations

VQ: VQ motif-containing
qRT-PCR: Quantitative real-time PCR
HMM: Hidden Markov Model
PI: Isoelectric point
II: Instability index
GRAVY: Grand average of hydropathicity
PEG 6000: Polyethylene glycol 6000
LT: Low temperature
HT: High temperature
MeJA: Methyl jasmonate
SA: Salicylic acid
ABA: Abscisic acid

## References

[1] Shewry PR, Hey SJ (2016). Do we need to worry about eating wheat?. Nutr Bull.

[2] Shewry PR (2009). Wheat. J Exp Bot.

[3] Shewry PR, Hey SJ (2015). The contribution of wheat to human diet and health. Food Energy Secur.

[4] Nakashima K, Yamaguchi-Shinozaki K. Promoters and transcription factors in abiotic stress-responsive gene expression. In: Abiotic stress adaptation in plants. Springer. 2009;199–216. 10.1007/978-90-481-3112-9_10.

[5] Baillo EH, Kimotho RN, Zhang Z, Xu P (2019). Transcription factors associated with abiotic and biotic stress tolerance and their potential for crops improvement. Genes (Basel).

[6] Gahlaut  V, Jaiswal  V, Kumar  A, Gupta  PK (2016). Transcription factors involved in drought tolerance and their possible role in developing drought tolerant cultivars with emphasis on wheat (*Triticum aestivum* L.). Theor Appl Genet.

[7] Wani  SH, Tripathi P, Zaid  A , Challa  GS, Kumar  A, Kumar  V (2018). Transcriptional regulation of osmotic stress tolerance in wheat (*Triticum aestivum* L.). Plant Mol Biol.

[8] Ulker B, Somssich IE (2004). WRKY transcription factors: from DNA binding towards biological function. Curr Opin Plant Biol.

[9] Weyhe M, Eschen-Lippold L, Pecher P, Scheel D, Lee J (2014). Menage a trois: the complex relationships between mitogen-activated protein kinases, WRKY transcription factors, and VQ-motif-containing proteins. Plant Signal Behav.

[10] Leon J, Gayubas B, Castillo MC (2020). Valine-glutamine proteins in plant responses to oxygen and nitric oxide. Front Plant Sci.

[11] Jing Y, Lin R. The VQ motif-containing protein family of plant-specific transcriptional regulators. Plant Physiol. 2015;169(1):371–8. 10.1104/pp.15.00788. 10.1104/pp.15.00788 PMC4577417 26220951

[12] Jiang SY, Sevugan M, Ramachandran S (2018). Valine-glutamine (VQ) motif coding genes are ancient and non-plant-specific with comprehensive expression regulation by various biotic and abiotic stresses. BMC Genomics.

[13] Cheng Y, Zhou Y, Yang Y, Chi YJ, Zhou J, Chen JY (2012). Structural and functional analysis of VQ motif-containing proteins in Arabidopsis as interacting proteins of WRKY transcription factors. Plant Physiol.

[14] Morikawa K, Shiina T, Murakami S, Toyoshima Y. Novel nuclear-encoded proteins interacting with a plastid sigma factor, Sig1, Arabidopsis thaliana. FEBS Lett. 2002;514(2–3):300–4. 10.1016/s0014-5793(02)02388-8. 10.1016/s0014-5793(02)02388-8 11943170

[15] Wang X, Zhang H, Sun G, Jin Y, Qiu L (2014). Identification of active VQ motif-containing genes and the expression patterns under low nitrogen treatment in soybean. Gene.

[16] Wang M, Vannozzi A, Wang G, Zhong Y, Corso M, Cavallini E (2015). A comprehensive survey of the grapevine VQ gene family and its transcriptional correlation with WRKY proteins. Front Plant Sci.

[17] Song W, Zhao H, Zhang X, Lei L, Lai J (2015). Genome-wide identification of VQ motif-containing proteins and their expression profiles under abiotic stresses in maize. Front Plant Sci.

[18] Wang A, Garcia D, Zhang H, Feng K, Chaudhury A, Berger F (2010). The VQ motif protein IKU1 regulates endosperm growth and seed size in Arabidopsis. Plant J.

[19] Lei R, Li X, Ma Z, Lv Y, Hu Y, Yu D (2017). Arabidopsis WRKY2 and WRKY34 transcription factors interact with VQ20 protein to modulate pollen development and function. Plant J.

[20] Li Y, Jing Y, Li J, Xu G, Lin R (2014). Arabidopsis VQ MOTIF-CONTAINING PROTEIN29 represses seedling deetiolation by interacting with PHYTOCHROME-INTERACTING FACTOR1. Plant Physiol.

[21] Hu Y, Chen L, Wang H, Zhang L, Wang F, Yu D (2013). Arabidopsis transcription factor WRKY8 functions antagonistically with its interacting partner VQ9 to modulate salinity stress tolerance. Plant J.

[22] Perruc E, Charpenteau M, Ramirez BC, Jauneau A, Galaud JP, Ranjeva R (2004). A novel calmodulin-binding protein functions as a negative regulator of osmotic stress tolerance in *Arabidopsis thaliana* seedlings. Plant J.

[23] Wang H, Hu Y, Pan J, Yu D (2015). *Arabidopsis* VQ motif-containing proteins VQ12 and VQ29 negatively modulate basal defense against *Botrytis cinerea*. Sci Rep.

[24] Ali MRM, Uemura T, Ramadan A, Adachi K, Nemoto K, Nozawa A (2019). The ring-type E3 ubiquitin ligase JUL1 targets the VQ-motif protein JAV1 to coordinate jasmonate signaling. Plant Physiol.

[25] Hu P, Zhou W, Cheng Z, Fan M, Wang L, Xie D (2013). JAV1 controls jasmonate-regulated plant defense. Mol Cell.

[26] Zou Z, Liu F, Huang S, Fernando WGD (2021). Genome-wide identification and analysis of the valine-glutamine motif-containing gene family in *Brassica napus* and functional characterization of *BnMKS1* in response to *Leptosphaeria maculans*. Phytopathology.

[27] Kim DY, Kwon SI, Choi C, Lee H, Ahn I, Park SR (2013). Expression analysis of rice VQ genes in response to biotic and abiotic stresses. Gene.

[28] Zhang C, Wang J, Long M, Fan C (2013). gKaKs: the pipeline for genome-level Ka/Ks calculation. Bioinformatics.

[29] Kumar D, Singh D, Kanodia P, Prabhu KV, Kumar M, Mukhopadhyay K. Discovery of novel leaf rust responsive microRNAs in wheat and prediction of their target genes. J Nucleic Acids. 2014;2014:570176. 10.1155/2014/570176. 10.1155/2014/570176 PMC4144313 25180085

[30] Han  R, Jian  C, Lv  J, Yan  Y, Chi  Q, Li  Z (2014). Identification and characterization of microRNAs in the flag leaf and developing seed of wheat (*Triticum aestivum* L.). BMC Genomics.

[31] Yao Y, Guo G, Ni Z, Sunkar R, Du J, Zhu JK (2007). Cloning and characterization of microRNAs from wheat  (*Triticum aestivum* L.). Genome Biol.

[32] Ramirez-Gonzalez RH, Borrill P, Lang D, Harrington SA, Brinton J, Venturini L, et al. The transcriptional landscape of polyploid wheat. Science. 2018;361(6403). 10.1126/science.aar6089. 10.1126/science.aar6089 30115782

[33] Eulgem T, Rushton PJ, Robatzek S, Somssich IE (2000). The WRKY superfamily of plant transcription factors. Trends Plant Sci.

[34] Buscaill P, Rivas S (2014). Transcriptional control of plant defence responses. Curr Opin Plant Biol.

[35] Wang Y, Jiang Z, Li Z, Zhao Y, Tan W, Liu Z, et al. Genome-wide identification and expression analysis of the VQ gene family in soybean (Glycine max). PeerJ. 2019;7:e7509. 10.7717/peerj.7509. 10.7717/peerj.7509 PMC6708371 31497394

[36] Liu C, Liu H, Zhou C, Timko MP. Genome-wide identification of the VQ protein gene family of tobacco (Nicotiana tabacum L.) and analysis of its expression in response to phytohormones and abiotic and biotic Stresses. Genes (Basel). 2020;11(3). 10.3390/genes11030284. 10.3390/genes11030284 PMC7140788 32156048

[37] Guo J, Chen J, Yang J, Yu Y, Yang Y, Wang W (2018). Identification, characterization and expression analysis of the VQ motif-containing gene family in tea plant (*Camellia sinensis*). BMC Genomics.

[38] Zhang G, Wang F, Li J, Ding Q, Zhang Y, Li H, et al. Genome-wide identification and analysis of the VQ motif-containing protein family in chinese cabbage (Brassica rapa L. ssp. Pekinensis). Int J Mol Sci. 2015;16(12):28683–704. 10.3390/ijms161226127. 10.3390/ijms161226127 PMC4691074 26633387

[39] Chu W, Liu B, Wang Y, Pan F, Chen Z, Yan H, et al. Genome-wide analysis of poplar VQ gene family and expression profiling under PEG, NaCl, and SA treatments. Tree Genet Genomes. 2016;12(6):124. 10.1007/s11295-016-1082-z.

[40] Ding H, Yuan G, Mo S, Qian Y, Wu Y, Chen Q (2019). Genome-wide analysis of the plant-specific VQ motif-containing proteins in tomato (*Solanum lycopersicum*) and characterization of SlVQ6 in thermotolerance. Plant Physiol Biochem.

[41] Dong Q, Zhao S, Duan D, Tian Y, Wang Y, Mao K (2018). Structural and functional analyses of genes encoding VQ proteins in apple. Plant Sci.

[42] Wang Y, Liu H, Zhu D, Gao Y, Yan H, Xiang Y (2017). Genome-wide analysis of VQ motif-containing proteins in Moso bamboo (*Phyllostachys edulis*). Planta.

[43] Moore RC, Purugganan MD (2003). The early stages of duplicate gene evolution. Proc Natl Acad Sci U S A.

[44] Storz JF (2009). Genome evolution: gene duplication and the resolution of adaptive conflict. Heredity (Edinb).

[45] Freeling M (2009). Bias in plant gene content following different sorts of duplication: tandem, whole-genome, segmental, or by transposition. Annu Rev Plant Biol.

[46] Wang Y, Wang X, Tang H, Tan X, Ficklin SP, Feltus FA (2011). Modes of gene duplication contribute differently to genetic novelty and redundancy, but show parallels across divergent angiosperms. PLoS ONE.

[47] Babbitt Cc, Haygood R, Wray Ga. When two is better than one. Cell. 2007;131(2):225–7. 10.1016/j.cell.2007.10.001. 10.1016/j.cell.2007.10.001 17956721

[48] Ohno S. Evolution by gene duplication. New York: Springer-Verlag; 1970. 10.2307/1530208.

[49] Luo M, Dennis ES, Berger F, Peacock WJ, Chaudhury A (2005). *MINISEED3* (*MINI3*), a *WRKY* family gene, and *HAIKU2* (*IKU2*), a leucine-rich repeat (*LRR*) KINASE gene, are regulators of seed size in *Arabidopsis*. Proc Natl Acad Sci U S A.

[50] Gargul JM, Mibus H, Serek M. Manipulation of MKS1 gene expression affects Kalanchoe blossfeldiana and Petunia hybrida phenotypes. Plant Biotechnol J. 2015;13(1):51–61. 10.1111/pbi.12234. 10.1111/pbi.12234 25082411

[51] Chen P, Wei F, Cheng S, Ma L, Wang H, Zhang M (2020). A comprehensive analysis of cotton VQ gene superfamily reveals their potential and extensive roles in regulating cotton abiotic stress. BMC Genomics.

[52] Pan J, Wang H, Hu Y, Yu D (2018). Arabidopsis VQ18 and VQ26 proteins interact with ABI5 transcription factor to negatively modulate ABA response during seed germination. Plant J.

[53] Zhu H, Zhou Y, Zhai H, He S, Zhao N, Liu Q. A novel sweetpotato WRKY transcription factor, IbWRKY2, positively regulates drought and salt tolerance in transgenic Arabidopsis. Biomolecules. 2020;10(4). 10.3390/biom10040506. 10.3390/biom10040506 PMC7226164 32230780

[54] Cheng X, Wang Y, Xiong R, Gao Y, Yan H, Xiang Y (2020). A Moso bamboo gene *VQ28* confers salt tolerance to transgenic Arabidopsis plants. Planta.

[55] Dong Q, Duan D, Zheng W, Huang D, Wang Q, Yang J (2022). Overexpression of *MdVQ37* reduces drought tolerance by altering leaf anatomy and SA homeostasis in transgenic apple. Tree Physiol.

[56] Yan H, Wang Y, Hu B, Qiu Z, Zeng B, Fan C (2019). Genome-wide characterization, evolution, and expression profiling of VQ gene family in response to phytohormone treatments and abiotic stress in *Eucalyptus grandis*. Int J Mol Sci.

[57] Bari R, Jones JD (2009). Role of plant hormones in plant defence responses. Plant Mol Biol.

[58] Ye YJ, Xiao YY, Han YC, Shan W, Fan ZQ, Xu QG (2016). Banana fruit VQ motif-containing protein5 represses cold-responsive transcription factor MaWRKY26 involved in the regulation of JA biosynthetic genes. Sci Rep.

[59] Dong Q, Duan D, Zheng W, Huang D, Wang Q, Li X, et al. MdVQ37 overexpression reduces basal thermotolerance in transgenic apple by affecting transcription factor activity and salicylic acid homeostasis. Hortic Res. 2021;8(1):220. 10.1038/s41438-021-00655-3. 10.1038/s41438-021-00655-3 PMC8484266 34593787

[60] Andreasson E, Jenkins T, Brodersen P, Thorgrimsen S, Petersen NH, Zhu S (2005). The MAP kinase substrate MKS1 is a regulator of plant defense responses. EMBO J.

[61] Jiang Y, Yu D (2016). The WRKY57 transcription factor affects the expression of jasmonate ZIM-domain genes transcriptionally to compromise *Botrytis cinerea* resistance. Plant Physiol.

[62] Lai Z, Li Y, Wang F, Cheng Y, Fan B, Yu JQ (2011). *Arabidopsis* sigma factor binding proteins are activators of the WRKY33 transcription factor in plant defense. Plant Cell.

[63] Zhang H, Zhang L, Ji Y, Jing Y, Li L, Chen Y (2021). Arabidopsis SIGMA FACTOR BINDING PROTEINs function antagonistically to WRKY75 in abscisic acid-mediated leaf senescence and seed germination. J Exp Bot.

[64] Bolser DM, Kerhornou A, Walts B, Kersey P (2015). Triticeae resources in ensembl plants. Plant Cell Physiol.

[65] Mistry J, Chuguransky S, Williams L, Qureshi M, Salazar GA, Sonnhammer ELL (2021). Pfam: The protein families database in 2021. Nucleic Acids Res.

[66] Marchler-Bauer A, Derbyshire MK, Gonzales NR, Lu S, Chitsaz F, Geer LY, et al. CDD: NCBI’s conserved domain database. Nucleic Acids Res. 2015;43:D222-226. 10.1093/nar/gku1221. 10.1093/nar/gku1221 PMC4383992 25414356

[67] Letunic I, Khedkar S, Bork P (2021). SMART: recent updates, new developments and status in 2020. Nucleic Acids Res.

[68] Wilkins MR, Gasteiger E, Bairoch A, Sanchez JC, Williams KL, Appel RD (1999). Protein identification and analysis tools in the ExPASy server. Methods Mol Biol.

[69] Garcia-Hernandez M, Berardini TZ, Chen G, Crist D, Doyle A, Huala E (2002). TAIR: a resource for integrated *Arabidopsis* data. Funct Integr Genomics.

[70] Goodstein DM, Shu S, Howson R, Neupane R, Hayes RD, Fazo J, et al. Phytozome: a comparative platform for green plant genomics. Nucleic Acids Res. 2012;40:D1178-1186. 10.1093/nar/gkr944. 10.1093/nar/gkr944 PMC3245001 22110026

[71] Kumar S, Stecher G, Tamura K. MEGA7: molecular evolutionary genetics analysis version 7.0 for bigger datasets. Mol Biol Evol. 2016;33(7):1870–4. 10.1093/molbev/msw054. 10.1093/molbev/msw054 PMC8210823 27004904

[72] Subramanian B, Gao S, Lercher MJ, Hu S, Chen WH (2019). Evolview v3: a webserver for visualization, annotation, and management of phylogenetic trees. Nucleic Acids Res.

[73] Hu B, Jin J, Guo AY, Zhang H, Luo J, Gao G. GSDS 2.0: an upgraded gene feature visualization server. Bioinformatics. 2015;31(8):1296–7. 10.1093/bioinformatics/btu817. 10.1093/bioinformatics/btu817 PMC4393523 25504850

[74] Bailey TL, Johnson J, Grant CE, Noble WS (2015). The MEME Suite. Nucleic Acids Res.

[75] Alaux M, Rogers J, Letellier T, Flores R, Alfama F, Pommier C (2018). Linking the International Wheat Genome Sequencing Consortium bread wheat reference genome sequence to wheat genetic and phenomic data. Genome Biol.

[76] Voorrips RE (2002). MapChart: software for the graphical presentation of linkage maps and QTLs. J Hered.

[77] Altschul SF, Madden TL, Schaffer AA, Zhang J, Zhang Z, Miller W (1997). Gapped BLAST and PSI-BLAST: a new generation of protein database search programs. Nucleic Acids Res.

[78] Gu Z, Cavalcanti A, Chen FC, Bouman P, Li WH. Extent of gene duplication in the genomes of Drosophila, nematode, and yeast. Mol Biol Evol. 2002;19(3):256–62. 10.1093/oxfordjournals.molbev.a004079. 10.1093/oxfordjournals.molbev.a004079 11861885

[79] Krzywinski M, Schein J, Birol I, Connors J, Gascoyne R, Horsman D (2009). Circos: an information aesthetic for comparative genomics. Genome Res.

[80] Wang D, Zhang Y, Zhang Z, Zhu J. KaKs_Calculator 2.0: a toolkit incorporating gamma-series methods and sliding window strategies. Genomics Proteomics Bioinformatics. 2010;8(1):77–80. 10.1016/S1672-0229(10)60008-3. 10.1016/S1672-0229(10)60008-3 PMC5054116 20451164

[81] Beier S, Thiel T, Munch T, Scholz U, Mascher M (2017). MISA-web: a web server for microsatellite prediction. Bioinformatics.

[82] Dai X, Zhuang Z, Zhao PX (2018). psRNATarget: a plant small RNA target analysis server (2017 release). Nucleic Acids Res.

[83] Shannon P, Markiel A, Ozier O, Baliga NS, Wang JT, Ramage D (2003). Cytoscape: a software environment for integrated models of biomolecular interaction networks. Genome Res.

[84] Chen C, Chen H, Zhang Y, Thomas HR, Frank MH, He Y (2020). TBtools: an integrative toolkit developed for interactive analyses of big biological data. Mol Plant.

[85] Lescot M, Dehais P, Thijs G, Marchal K, Moreau Y, Van de Peer Y (2002). PlantCARE, a database of plant cis-acting regulatory elements and a portal to tools for in silico analysis of promoter sequences. Nucleic Acids Res.

[86] Han Z, Liu Y, Deng X, Liu D, Liu Y, Hu Y (2019). Genome-wide identification and expression analysis of expansin gene family in common wheat  (*Triticum aestivum* L.). BMC Genomics.

[87] Liu H, Xing M, Yang W, Mu X, Wang X, Lu F (2019). Genome-wide identification of and functional insights into the late embryogenesis abundant (LEA) gene family in bread wheat (*Triticum aestivum*). Sci Rep.

[88] Duan YH, Guo J, Ding K, Wang SJ, Zhang H, Dai XW (2011). Characterization of a wheat HSP70 gene and its expression in response to stripe rust infection and abiotic stresses. Mol Biol Rep.

[89] Su HG, Zhang XH, Wang TT, Wei WL, Wang YX, Chen J (2020). Genome-wide identification, evolution, and expression of GDSL-type esterase/lipase gene family in soybean. Front Plant Sci.

[90] Zhao W, Zhang LL, Xu ZS, Fu L, Pang HX, Ma YZ, et al. Genome-wide analysis of MADS-Box genes in foxtail millet (Setaria italica L.) and functional assessment of the role of SiMADS51 in the drought stress response. Front Plant Sci. 2021;12:659474. 10.3389/fpls.2021.659474. 10.3389/fpls.2021.659474 PMC8273297 34262576

[91] Szklarczyk D, Gable AL, Nastou KC, Lyon D, Kirsch R, Pyysalo S (2021). The STRING database in 2021: customizable protein-protein networks, and functional characterization of user-uploaded gene/measurement sets. Nucleic Acids Res.

